# Choroidal hemodynamics in central serous chorioretinopathy after half-dose photodynamic therapy and the effects of smoking

**DOI:** 10.1038/s41598-022-21584-8

**Published:** 2022-10-11

**Authors:** Etsuyo Horiguchi, Jun Takeuchi, Ryo Tomita, Keiko Asai, Yuyako Nakano, Hikaru Ota, Yosuke Taki, Yasuki Ito, Hiroko Terasaki, Koji M. Nishiguchi, Keiko Kataoka

**Affiliations:** 1 Department of Ophthalmology, Nagoya University Graduate School of Medicine, Nagoya, Aichi Japan; 2 Department of Ophthalmology, Kyorin University School of Medicine, 6-20-2, Shinkawa, Mitaka-shi, Tokyo, Japan; 3 Department of Ophthalmology, Fujita Health University, Toyoake, Aichi Japan

**Keywords:** Macular degeneration, Retinal diseases

## Abstract

This retrospective study aimed to evaluate choroidal hemodynamics after half-dose photodynamic therapy (PDT) for central serous chorioretinopathy (CSC) and the effects of smoking using laser speckle flowgraphy. This study included 29 eyes of 29 patients treated with half-dose PDT for CSC, who were followed-up for at least 6 months. The mean blur rate (MBR) in the PDT irradiation area (whole area), the pachyvessel (PV) area, non-PV (NPV) area, and filling delay (FD) area were assessed at baseline and 1, 3, and 6 months post-PDT, respectively. The MBR was also assessed by smoking status. The MBR significantly decreased from baseline in the whole, PV, NPV, and FD areas at all time points (*P* < 0.001). Of the 29 patients, 6 were never smokers, 13 were past smokers, and 10 were current smokers. At baseline, no significant difference was found in the MBR in the whole, PV, NPV, and FD areas among never, past, and current smokers. The MBR changes showed a significantly smaller decrease in current smokers than in never smokers in the whole (*P* = 0.021), PV (*P* = 0.009), and NPV (*P* = 0.034) areas, but not in the FD area (*P* = 0.172). Half-dose PDT for CSC reduced choroidal blood flow in the PDT-irradiated area, which was blunted by current smoking status.

## Introduction

Central serous chorioretinopathy (CSC) is a relatively common disease among middle-aged people characterized by serous retinal detachment. Although the exact pathogenesis of CSC is not completely understood, the current understanding of its mechanisms involves choroidal vascular hyperpermeability following venous overload^1^. When serous retinal detachment persists or recurs, vision is irreversibly threatened due to damage to the photoreceptor layer. Safety-enhanced verteporfin photodynamic therapy (PDT), such as half-dose, half-fluence, and half-time PDT has been reported to lead to the rapid absorption of subretinal fluid and has been widely used in the present decade^2–6^. Recent studies of the choroid using optical coherence tomography (OCT) revealed that safety-enhanced PDT for chronic CSC induces subfoveal choroidal thinning and notably decreases the area of choroidal lumens^7–9^. However, the change in macular hemodynamics related to the safety-enhanced PDT for chronic CSC has not yet been thoroughly discussed. As an example, smoking is a factor that can cause choroidal hemodynamic changes. Furthermore, smoking is a risk factor associated with CSC, in addition to stress and steroid usage, suggesting that it may be related to the pathophysiology of CSC^10^.

Laser speckle flowgraphy (LSFG) is a novel imaging modality that allows noninvasive quantitative measurement of choroidal blood flow as a relative index of blood flow velocity and mean blur rate (MBR). Recent studies using LSFG showed that MBR and vascular resistance decreased concurrently with the resolution of serous retinal detachment in eyes with acute CSC^11^. However, little is known about hemodynamics before and after treatment for chronic CSC. In this study, we investigated macular hemodynamics after half-dose PDT for CSC using LSFG. Furthermore, given the aforementioned influence of smoking, we compared hemodynamics after half-dose PDT between smokers and nonsmokers.

## Results

**Figure 1:**
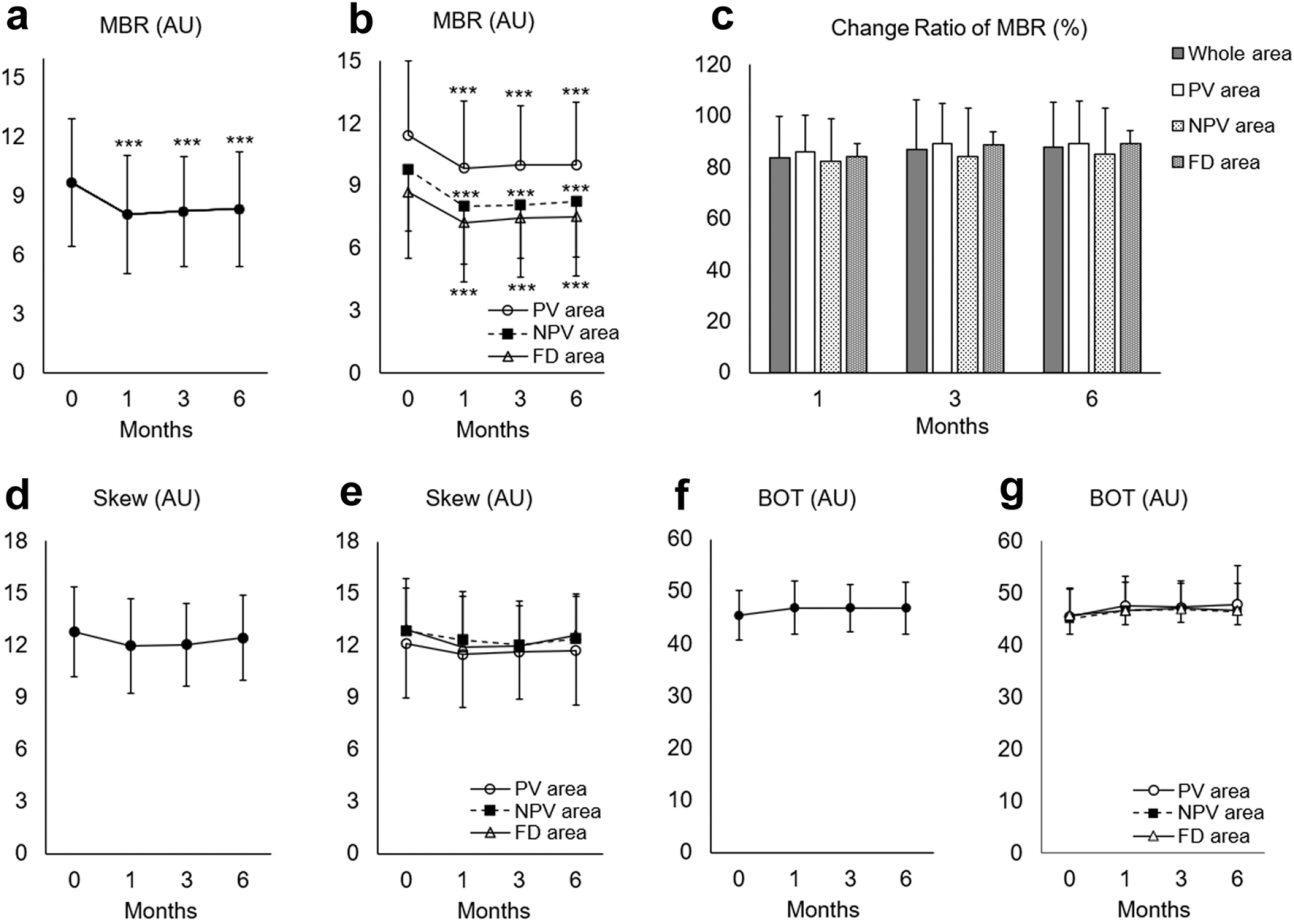
Change of mean blur rate (MBR), skew, blowout time (BOT) after half-dose photodynamic therapy (PDT). (**a**) The MBR of the PDT irradiation area was significantly decreased from baseline after half-dose PDT. (**b**) The MBR in the pachyvessel area (PV area), the area without pachyvessels or filling delay (NPV area), and the filling delay area (FD area) significantly decreased from baseline. (**c**) The changes in the MBR from baseline were not significantly different among the PV, NPV, and FD areas at 1, 3, and 6 months. (**d**) The skew in the whole area. (**e**) The skew in the PV, NPV, and FD areas. (**f**) The BOT in the whole area. (**g**) The BOT in the PV, NPV, and FD areas. AU: arbitrary units. ***P < 0.001.

A total of 29 eyes of 29 patients (23 men and 6 women; mean age, 57.2 ± 11.1 years) treated with half-dose PDT for CSC were included. The baseline characteristics of the patients are shown in Table 1. The logarithm of the minimum angle of resolution best-corrected visual acuity (logMAR BCVA) was 0.18 ± 0.20 (mean Snellen visual acuity, 20/30; range, 20/100–20/20). Among the 29 patients, 6 were never smokers, 13 were past smokers, and 10 were current smokers. Among the past and current smokers, the mean number of cigarettes smoked per day was 20 ± 13.5 (22 ± 15.5 for past smokers and 16 ± 9.9 for current smokers), and the mean duration of smoking was 28 ± 12.6 years (25 ± 14.8 years for past smokers and 32 ± 8.1 years for current smokers). The mean Brinkman index, which is defined as the number of cigarettes smoked per day multiplied by the number of years of smoking, was 526 ± 321.7 (549 ± 368.0 for past smokers and 496 ± 265.8 for current smokers). Among the 29 eyes, 28 eyes presented diffuse leakage on fluorescein angiography (FA) and wide spread retinal pigment epithelial (RPE) disturbance on fundus autofluorescence (FAF); one eye, which showed recurrent and long-lasting subretinal fluid for more than 6 months, presented focal leakage with only small RPE disturbance on FAF. The average MBR in the whole area was 9.7 ± 3.3 at baseline. After half-dose PDT, the average MBR significantly decreased to 8.1 ± 3.0 at 1 month, 8.2 ± 2.8 at 3 months, and 8.3 ± 2.9 at 6 months (Fig. 1a). When assessing each area, such as the pachyvessel (PV) area, non-pachyvessel (NPV) area, and filling delay (FD) areas, the MBR in all three areas significantly decreased from those at baseline at 1, 3, and 6 months (Fig. 1b). Compared to the PV area, the MBR in the FD area was significantly lower at baseline and at 1, 3, and 6 months (*P* = 0.011, *P* = 0.007, *P* = 0.005, *P* = 0.009, respectively). The MBR in the NPV area at 3 months was significantly lower than that of the PV area (*P* = 0.034). The change ratio of the MBR from baseline was not significantly different among the PV, NPV, and FD areas at 1, 3, and 6 months (*P* = 0.709, *P* = 0.578, and *P* = 0.631, respectively, Fig. 1c). The skew did not change over the 6-month follow-up period in the whole, PV, NPV, and FD areas (*P* = 0.295, *P* = 0.704, *P* = 0.386, *P* = 0.241, respectively, Fig. 1d and e). Similarly, the blow out time (BOT) did not change over the 6-month follow-up period in the whole, PV, NPV, and FD areas (*P* = 0.246, *P* = 0.188, *P* = 0.135, *P* = 0.675, respectively, Fig. 1f and g).

**Table 1:** Baseline characteristics of patients.

Age, years	57.2 ± 11.1
Male/Female, eyes	23/6
LogMAR BCVA	0.18 ± 0.20
Axial length, mm	24.2 ± 1.1
IOP, mmHg	14.8 ± 3.2
OPP, mmHg	54.4 ± 7.2
History of Smoking, Never/Past/Current, eyes	6/13/10
Duration from onset to PDT, months	22.6 ± 56.4

*LogMAR* logarithm of the minimum angle of resolution, *BCVA* best-corrected visual acuity, *IOP* intraocular pressure, *OPP* ocular perfusion pressure, *PDT* photodynamic therapy. Data are indicated as mean ± standard deviation.

**Figure 2:**
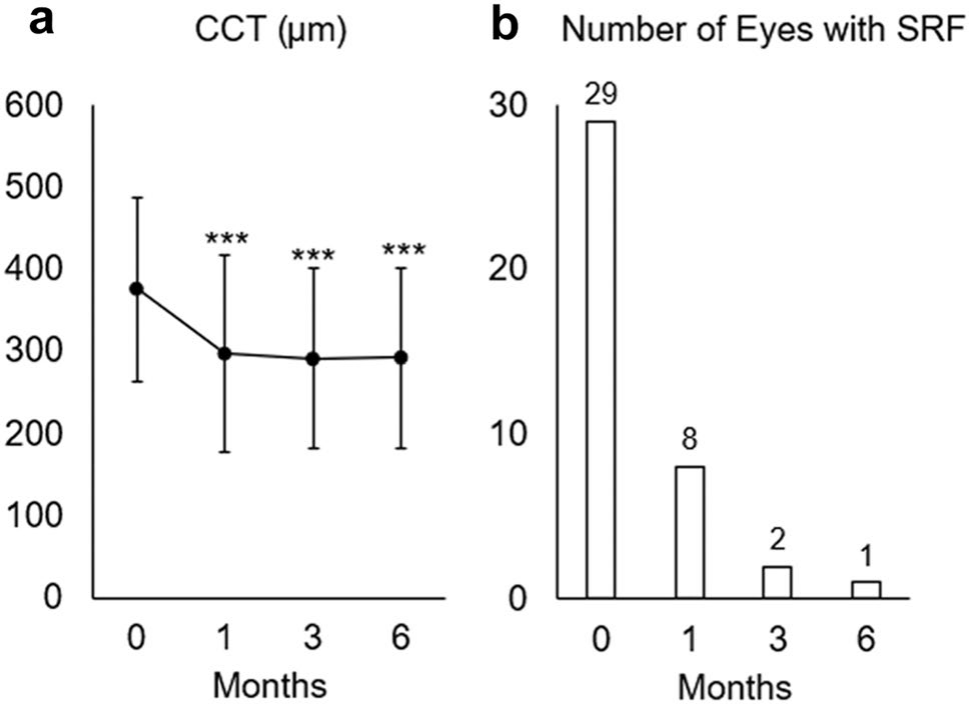
Change of central choroidal thickness (CCT) and the presence of subretinal fluid (SRF) after half-dose photodynamic therapy (PDT). (**a**) The CCT significantly decreased after half-dose PDT. ***P < 0.001. (**b**) The SRF was resolved after half-dose PDT; one case showed residual SRF at 6 months.

The average central choroidal thickness (CCT) was significantly decreased from 376.0 μm at baseline to 296.8 μm at 1 month, 290.8 μm at 3 months, and 292.4 μm at 6 months (Fig. 2a). Regarding subretinal fluid, 28 eyes showed complete resolution of the subretinal fluid by 6 months. One eye showed residual subretinal fluid over the 6-month follow-up period, although it was partially resolved (Fig. 2b). Table 2 shows the breakdown of eyes with residual subretinal fluid by smoking status. It is noted that subretinal fluid in never smokers was completely absorbed by 1 month. The smoking status significantly affected the speed of subretinal fluid resolution. No significant difference in the change ratio of MBR was observed between eyes with or without complete resolution of residual subretinal fluid at 1 month. No correlation was observed between MBR and CCT changes from baseline at 1 month, 3 months, or 6 months, respectively (*P* = 0.068, *P* = 0.083, *P* = 0.159, Pearson’s correlation coefficient).

**Table 2:** Residual subretinal fluid and smoking status.

	Baseline	1 month	3 months	6 months	P*
Never smoker, eyes	6	0	0	0	
Past smoker, eyes	13	3	1	0	0.10^a^
Current smoker, eyes	10	5	1	1	
Total number, eyes	29	8	2	1	

^a^Fisher's exact test.

Of the 29 patients, 9 received concurrent half-dose PDT for the fellow eye. Thus, the data of the remaining 20 patients were used for the comparisons of MBR between the treated and untreated fellow eyes. The MBR in the treated eyes was significantly decreased compared to that in the fellow eyes at 1 month after half-dose PDT, but there was no significant difference in the MBR between the treated eyes and fellow eyes at baseline, 3 months, and 6 months (Supplementary Figure S1a). The CCT in the treated eyes was significantly thicker than that in the fellow eyes at baseline, but no significant differences in CCT between the treated and fellow eyes were observed after half-dose PDT (Supplementary Figure S1b).

The average mean ocular perfusion pressure (MOPP) was 54.4 mmHg at baseline and was significantly decreased at 1 month (50.9 mmHg, *P* = 0.008) and 6 months (51.2 mmHg, *P* = 0.016), but not significantly different at 3 months (51.7 mmHg, *P* = 0.056). The average mean arterial blood pressure (MAP) also decreased from 103.8 mmHg at baseline to 99.5 mmHg at 1 month (*P* = 0.069), 99.1 mmHg (*P* = 0.041) at 3 months, and 98.5 mmHg at 6 months (*P* = 0.015), although the mean IOP did not show significant changes during the follow-up period (14.8 mmHg at baseline, 15.4 mmHg at 1 month, 15.2 mmHg at 3 months, and 14.5 mmHg at 6 months, repeated analysis of variance (ANOVA), *P* = 0.326). The Pearson correlation analysis showed no significant correlation between the baseline MBR and baseline MOPP (r = 0.364, *P* = 0.052) and the change ratio of MBR and that of MOPP at 1, 3, and 6 months (r = -0.09, *P* = 0.63; r = 0.08, *P* = 0.68; r = 0.20, *P* = 0.30, respectively).

Next, we divided the 29 eyes into two groups according to the MBR change at 1 month after the treatment: The small decrease group included eyes with an MBR decrease ≤ 20% and the large decrease group included those with an MBR decrease > 20%. The small decrease group included higher percentages of past and current smokers and showed thicker CCT at baseline than the large decrease group (Table 3). However, there were no significant differences in age, sex, logMAR BCVA at baseline, axial length, CCT change at 1 month, MBR at baseline, greatest linear dimension of half-dose PDT, and duration from the onset of CCT to half-dose PDT between the two groups.

**Table 3:** Comparisons of the Change in MBR and Clinical Characteristics.

	Small decrease group (n = 18)	Large decrease group (n = 11)	*P*
Age, years	54.2 ± 7.0	62.1 ± 14.8	0.12^a^
Male, eyes (%)	15 (83.8)	8 (72.7)	0.41^b^
LogMAR BCVA at baseline	0.18 ± 0.24	0.17 ± 0.13	0.49^c^
AL, mm	24.2 ± 0.8	24.3 ± 1.5	0.73^a^
Smoking status, Never/past/current smokers, eyes	0/9/9	6/4/1	< 0.001^b^
CCT at baseline, μm	422.2 ± 105.4	300.4 ± 77.6	0.003^a^
CCT change at one month, %	82.1 ± 13.7	71.0 ± 19.8	0.085^a^
MBR at baseline, AU	10.1 ± 3.5	9.0 ± 2.8	0.41^a^
GLD of half-dose PDT, μm	4356.8 ± 816.0	4391.5 ± 920.9	0.92^a^
Duration from onset to PDT	9.5 ± 7.7	30.5 ± 70.9	0.25^c^

*MBR* mean blur rate, *LogMAR* logarithm of the minimum angle of resolution, *BCVA* best-corrected visual acuity, *AL* axial length, *CCT* central choroidal thickness, *GLD* greatest linear dimension, *PDT* photodynamic therapy. a: Student’s t-test. b: chi-square test. c: Mann–Whitney U test. Data are indicated as mean ± standard deviation.

**Figure 3:**
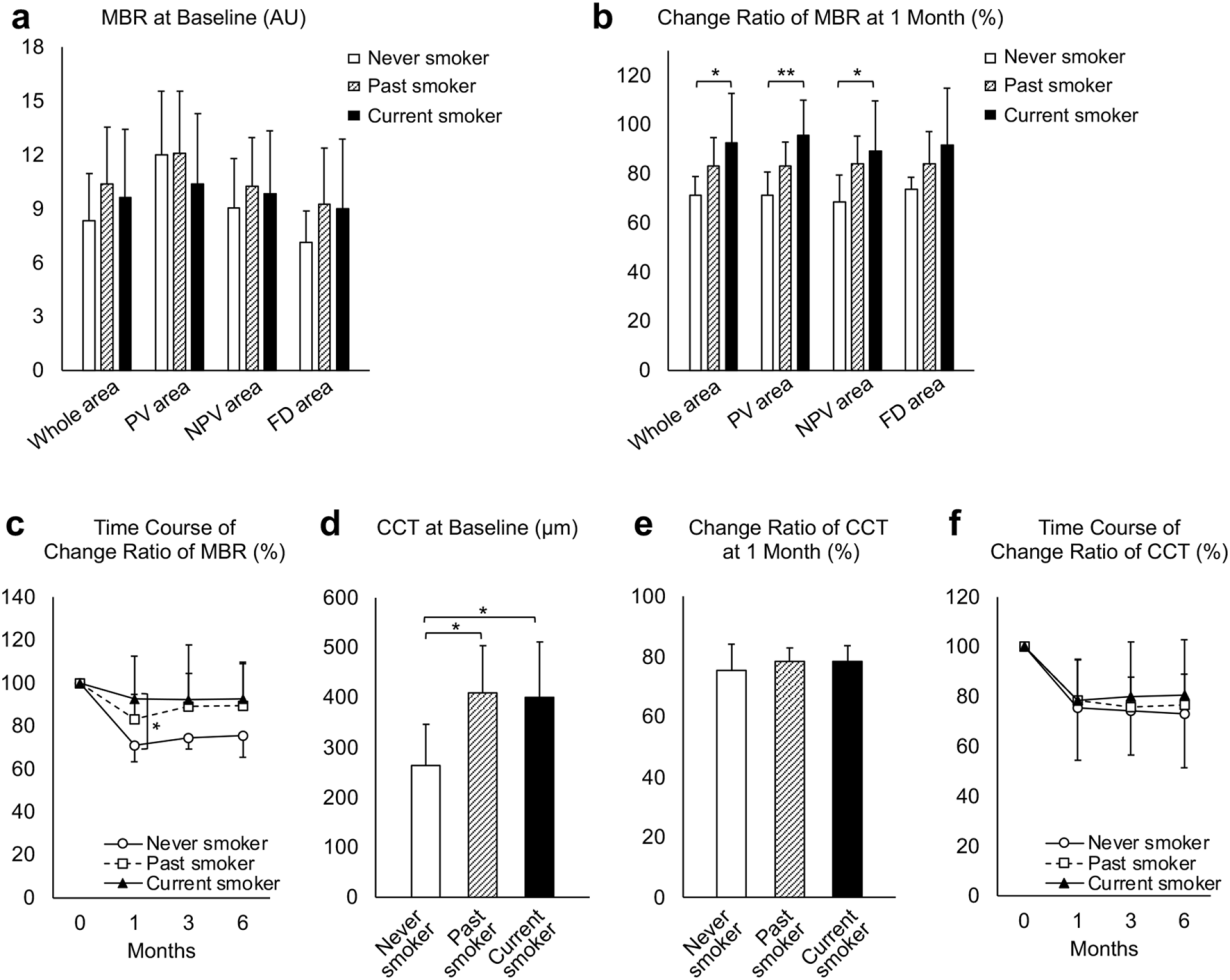
Analyses of mean blur rate (MBR) and central choroidal thickness (CCT) according to smoking status. (**a**) The MBR of the half-dose photodynamic therapy (PDT) irradiation area (whole area), the pachyvessel area (PV area), the area without pachyvessels or filling delay (NPV area), and the filling delay area (FD area) showed no significant difference among never smokers, past smokers, and current smokers. (**b**) The change ratio of MBR from baseline at 1 month after half-dose PDT showed that the MBR decrease in current smokers was significantly smaller compared to that in never smokers in the whole, PV, and NPV areas, but not in the FD area. (**c**) The mean MBR decreases in current and past smokers were smaller than those in never smokers and were not significant at 3 and 6 months. (**d**) The CCT at baseline was significantly thicker in past and current smokers compared to that in never smokers. (**e**) The change in CCT at 1 month showed no significant difference among never smokers, past smokers, and current smokers. AU: arbitrary units. *P < 0.05, **P < 0.01. (**f**) CCT decreases at 1, 3, and 6 months did not significantly correlate with smoking status.

**Figure 4:**
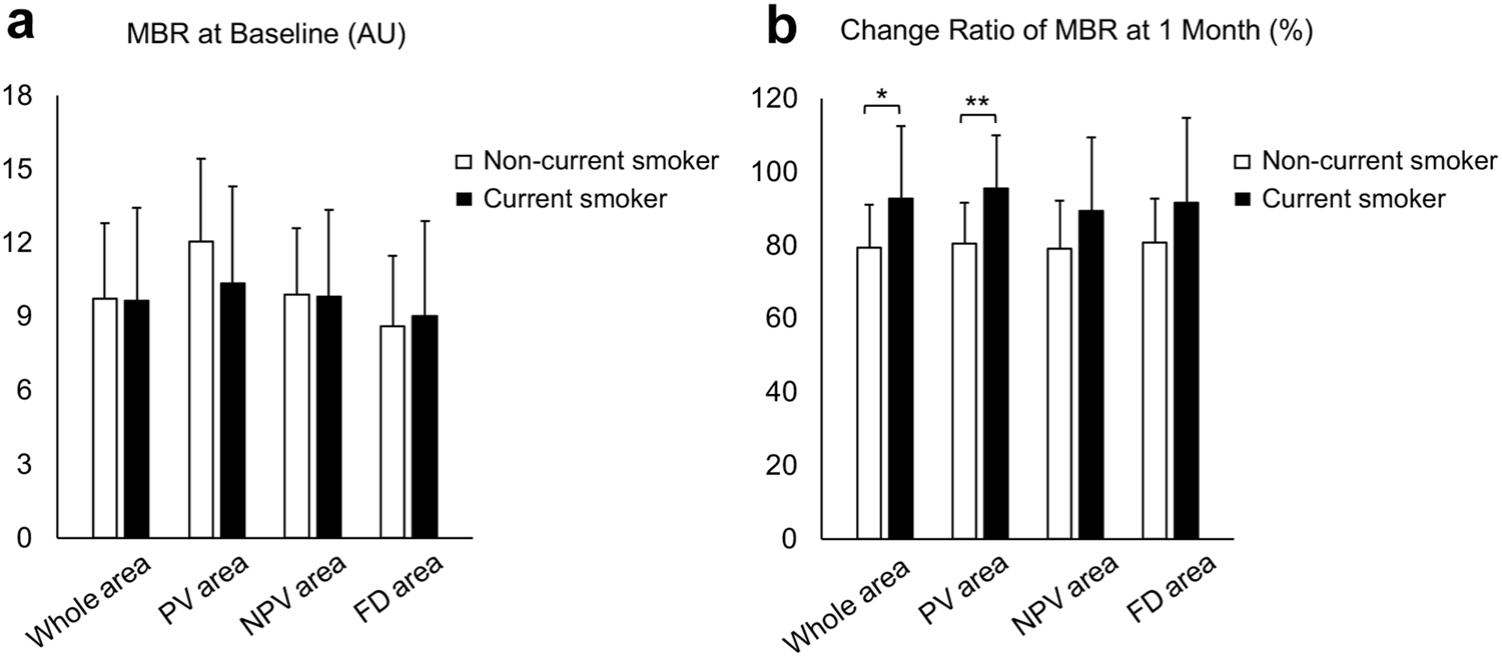
Analyses of the mean blur rate (MBR) according to current smoking status. (**a**) The MBR of the half-dose photodynamic therapy (PDT) irradiation area (whole area), the pachyvessel area (PV area), the area without pachyvessels or filling delay (NPV area), and the filling delay area (FD area) showed no significant difference between non-current smokers and current smokers. (**b**) The change ratio of MBR from baseline at 1 month after half-dose PDT showed that the MBR decrease in current smokers was significantly smaller compared with that in non-current smokers in the whole and PV area, but not in the NPV nor FD area. AU: arbitrary units. *P < 0.05, **P < 0.01.

We then analyzed the effects of smoking on the choroid. Compared with the MBR in the whole, PV, NPV, and FD areas at baseline, there was no significant difference among never smokers, past smokers, and current smokers (Fig. 3a). With regard to the MBR changes 1 month after half-dose PDT, the MBR changes of current smokers were significantly smaller compared to those of never smokers in the whole (*P* = 0.021), PV (*P* = 0.009), and NPV (*P* = 0.034) areas, but not in the FD area (*P* = 0.172) (Fig. 3b). A similar trend was observed in MBR changes at 3 and 6 months, but this trend was not significantly correlated with smoking status (Fig. 3c). The percentages of PV, NPV, and FD areas were 11.2 ± 8.2%, 34.3 ± 31.6%, and 46.2 ± 28.3% in never smokers, 16.2 ± 7.2%, 32.0 ± 23.0%, and 36.7 ± 17.1% in past smokers, and 10.2 ± 4.8%, 44.8 ± 28.2%, and 26.5 ± 22.0% in current smokers, respectively. No significant correlation was observed in the percentage of PV area (*P* = 0.100), NPV area (*P* = 0.513), and FD area (*P* = 0.277) and smoking status. At baseline, the CCT in never smokers was significantly lower than that in past and current smokers (*P* = 0.017 and *P* = 0.032, respectively; Fig. 3d). The CCT changes at 1 month after half-dose PDT showed no significant difference among never smokers, past smokers, and current smokers (*P* = 0.936, Fig. 3e); this was similar at 3 and 6 months (Fig. 3f). With regard to the total choroidal, luminal, and stromal areas, there was no significant difference at baseline and in any changes of the analyzed areas at 1 month according to smoking status (Supplementary Fig. S2a and b). The choroidal vascular index (CVI) at baseline and the change in CVI at 1 month also did not significantly differ according to smoking status (Supplementary Figure S2c and d). When the patients were divided into two groups; non-current smokers, including never smokers and past smokers (n = 19), and current smokers (n = 10), the MBR in the whole, PV, NPV, and FD area at baseline showed no significant difference between the non-current smokers and current smokers (Fig. 4a). However, the current smokers showed significantly smaller MBR changes at 1 month compared to non-current smokers in the whole area (*P* = 0.032) and PV area (*P* = 0.005), but not in the NPV area (*P* = 0.113) and FD area (*P* = 0.146, Student’s *t*-test, Fig. 4b). Among past and current smokers, there was no significant correlation between Brinkman index and CCT at baseline (*P* = 0.462, Pearson’s correlation coefficient), CCT changes at 1 month (*P* = 0.596, Pearson’s correlation coefficient), CVI at baseline (P = 0.120, Pearson’s correlation coefficient), or CVI change at 1 month (*P* = 0.881, Pearson’s correlation coefficient).

## Discussion

The pathogenesis of chronic CSC remains controversial. Recent structural analysis of the choroid using OCT has revealed that CSC-affected eyes have a thicker sclera, which may increase choroidal outflow resistance and choroidal congestion in vortex veins^12^. It has been speculated that the choroidal venous overload underlying CSC may lead to changes in hemodynamic and vascular morphology because, in addition to the thick choroid, choroidal veins are dilated and anastomose at the watershed zone^1,13–16^. In acute CSC eyes, MBR was reported to increase at the onset of CSC and decrease concordantly with the regression of subretinal fluid, suggesting that choroidal hyperperfusion, probably due to increased sympathetic activity, is related to the pathogenesis of the disease^11,17–19^. On the other hand, chronic CSC is a condition in which the subretinal fluid persists without spontaneous absorption; thus, choroidal hemodynamics may not be necessarily the same as those of acute CSC.

PDT for CSC is still being used in many countries as an off-label use. Among several treatment methods including laser photocoagulation, micropulse laser, and anti-vascular endothelial growth factor agents^1^, safety-enhanced PDT is considered to be the most promising treatment for chronic CSC at the present time, targeting the area of choroidal hyperpermeability visualized by indocyanine green angiography (ICGA), and not the systemic hyperperfusion or thickened sclera. Therefore, the primary pathogenesis of chronic CSC is speculated to be shifting to a localized area of choroidal hyperpermeability and RPE disturbance associated with localized blood flow alteration, although a thickened sclera and choroidal hyperperfusion may trigger the onset of CSC. With regard to the choroidal hemodynamics after half-dose PDT for CSC, Kumashiro et al. reported that the macular MBR decreased after treatment; this effect was blunted by 1/3-dose PDT^20^. In the current study, the MBR in the PDT irradiation area was significantly decreased after half-dose PDT, which is consistent with the report by Kumashiro et al. In addition, the current study showed that the MBR did not change in the untreated fellow eyes. Thus, these data suggest that half-dose PDT for CSC decreases choroidal blood velocity in the irradiation area, but not systemically. The choroid comprises the choriocapillaris, medium-sized arterioles, and venules in Sattler’s layer and large choroidal veins in Haller’s layer^21^. The blood velocity in the choriocapillaris is extremely slow and has been reported to be approximately one-third of that of retinal capillaries, suggesting that the blood flow in the choriocapillaris probably does not affect LSFG measurements^22^. The current study showed that the average MOPP and MAP, but not IOP, were higher at baseline compared to other time points. MAP may have increased temporarily due to the anxiety of the patients before half-dose PDT, which may have receded after the treatment. Furthermore, sympathetic nerve system overactivity, one cause of CSC, may have presented and led to hypertension. No correlation between the baseline MBR and baseline MOPP suggests that the higher MOPP at baseline does not affect the interpretation of MBR data.

In the current study, we labeled the PV area according to the presence of large-sized choroidal veins in the mid-phase ICGA; thus, the rest of the areas, such as the NPV and FD areas, exhibited blood flow mainly from medium-sized arterioles and venules, whereas the PV area exhibited blood flow not only from medium-sized arterioles and venules, but also from large-sized choroidal veins. During the 6-month follow-up period, the MBR in the FD area was significantly lower than that in the PV area where choroidal filling was not delayed. The FD area was labelled according to the choroidal filling delay observed in the arterial phase of ICGA. Thus, a lower MBR in the FD area is consistent with insufficient blood flow into the choroidal arterioles. Interestingly, in the current study, the reduction of the MBR after half-dose PDT was comparable in the PV, NPV, and FD areas. PDT is known to induce damage to endothelial cells and vascular occlusion mainly in the choriocapillaris and, to some extent, medium-sized choroidal vessels, rather than in large-sized choroidal vessels^23,24^. Thus, we speculate that the data showing MBR reduction may indicate that the half-dose PDT reduces blood flow in medium-sized arterioles and venules, which are common in the PV, NPV, and FD areas. Subsequently, blood flow running into large-sized veins, such as pachyvessels, may be reduced, and the local choroidal overload may be resolved. Due to the dynamic change of choroidal blood flow after half-dose PDT, vascular resistance would be expected to change; however, the current study showed that both skew and BOT, which are indicators of vascular resistance, did not significantly change. We speculate that the significant decrease in the choroidal vascular lumen after half-dose PDT could cancel the change in vascular resistance. If patients had underwent LSFG measurements at an early time point after half-dose PDT, we could have detected a more dynamic change in blood flow resistance. Although the MBR was found to decrease after half-dose PDT, it cannot be drawn from the current findings whether the MBR of chronic CSC is higher than that in healthy eyes. In the present study, the comparison of the MBR in treated and untreated fellow eyes showed no significant difference at baseline. However, fellow eyes of unilateral CSC eyes are known to present pachychoroid characteristics^25^; thus, the MBR in the untreated fellow eyes may be different from that in healthy eyes. Further studies are required to assess whether the pathogenesis of chronic CSC is a result of choroidal congestion or choroidal hyperperfusion.

In the current study, we revealed that the change in MBR after half-dose PDT for chronic CSC was different between current smokers and never smokers. This is the first report to reveal that smoking affects macular hemodynamics after half-dose PDT. Smoking is known to be a risk factor for chronic CSC^10^. In healthy eyes, Kantarci et al. reported that choroidal thickness in long-term smokers and never smokers did not differ^26^. In addition, Wei et al. reported that the CVI in healthy smokers was smaller than that in healthy never smokers in a dose-dependent manner, although choroidal thickness did not differ between the two groups^27^. However, Okawa et al. reported that CSC eyes of past and current smokers showed thicker choroids than those of never smokers, but that there was no difference in the CVI^28^. Similarly to the previous report by Okawa et al., the current study showed that in eyes with chronic CSC, CCT in current smokers was thicker than that in never smokers, but there was no difference in the CVI. Furthermore, we revealed that current smokers showed a significantly smaller decrease in MBR after half-dose PDT compared to never smokers. On comparing the MBR change at 1 month between the current and non-current smokers, the current smokers showed a significantly smaller decrease in the whole area and PV area, suggesting that the habit of smoking may affect hemodynamics in the pachyvessels. In regards to the speed of subretinal fluid after half-dose PDT, all eyes with residual subretinal fluid at 1 month were those of past or current smokers. Although there was no significant change in the ratio of MBR at 1 month between eyes with and without residual subretinal fluid, the change of MBR or smoking status may affect the speed of subretinal fluid resolution. These sub-analyses based on smoking status should be considered a pilot study. Further large-scale studies are needed.

In the current study, some eyes showed widespread RPE atrophy. When the area for LSFG analyses overlapped with an atrophic area, the data from the atrophic area was excluded from the analyses, as described in the methods. Thus, the results were not affected by the atrophic areas.

The limitations of this study include the small sample size and the retrospective nature of the analysis with short follow-up periods. A limitation of the LSFG technology is the two-dimensional analysis of blood flow in the retina and choroid, although the macula MBR includes flow information mainly from the choroid^29^. The analysis of MBR in PV and NPV using the rubber band divided into squares with a side of approximately 240 μm was not precise enough to evaluate the hemodynamics between PV and NPV. A rubber band divided into smaller squares would be more suitable for a detailed analysis. We did not evaluate the change in choroidal hyperpermeability on ICGA after half-dose PDT; thus, this study did not assess the correlation between changes in LSFG and hyperpermeability.

In summary, this study revealed that half-dose PDT reduced choroidal blood flow velocity not only in the entire PDT irradiation area but also in each PV, NPV, and FD area within the PDT irradiation area, which may suggest the improvement of focal choroidal overload. Furthermore, smoking status blunted the change in choroidal dynamics after half-dose PDT. This effect of smoking is a novel finding and provides a clue to understanding the mechanisms of CSC.

## Methods

### Ethics statement

The present study was a retrospective observational case series. This study adhered to the tenets of the Declaration of Helsinki. The study protocol was approved by the Institutional Review Board of Nagoya University Graduate School of Medicine (2021-0219) and was appropriately registered with the University Hospital Medical Information Network (UMIN000046481). The institutional review board of Nagoya University Graduate School of Medicine waived the requirement for informed consent because of the retrospective nature of the study.

### Subjects

We collected data on consecutive patients diagnosed with chronic CSC who underwent half-dose PDT at Nagoya University Hospital between March 2019 and February 2021. All patients underwent BCVA testing, intraocular pressure (IOP) measurements with non-contact tonometry (TONOPACHY; NIDEC, Aichi, Japan), axial length measurements (IOLMaster 500; Carl Zeiss Meditec, Jena, Germany), enhanced depth imaging spectral-domain OCT in follow-up mode (Spectralis; Heidelberg Engineering, Heidelberg, Germany), FA/ICGA (Spectralis HRA + OCT; Heidelberg Engineering), swept-source OCT angiography (PlexElite/AngioPlex; Carl Zeiss Meditec), and LSFG (LSFG-NAVI; Softcare, Fukuoka, Japan). CSC was diagnosed in the presence of persistent serous retinal detachment for at least 3 months on OCT, associated with thick choroid and choroidal vascular hyperpermeability on ICGA. The exclusion criteria were eyes previously treated with laser photocoagulation, micropulse laser, oral medications with aldosterone antagonists, intravitreal injection of anti-vascular endothelial growth factor agents, or PDT. The presence of choroidal neovascularization was assessed using OCT angiography; eyes were excluded if choroidal neovascularization was present. Systolic blood pressure (SBP) and diastolic blood pressure (DBP) were measured using an automatic sphygmomanometer (HBP-9035; OMRON DALIAN, Kyoto, Japan) before the LSFG measurements. The MAP and MOPP were calculated.

$$ \begin{aligned} {\text{MAP }} = & {\text{ DBP }} + { 1}/{3}\left( {{\text{SBP }} - {\text{ DBP}}} \right) \\ {\text{MOPP }} = &\, { 2}/{\text{3MAP }} - {\text{ IOP}} \\ \end{aligned} $$

### Half-dose PDT

A half-dose of verteporfin (3 mg/m^2^, Visudyne; CLINIGEN K.K., Staffordshire, UK) was infused over 8 min, followed by delivery of a 689-nm diode laser at 10 min after the beginning of the infusion, following the procedures of a previous report^3^. A total of 50 J/cm^2^ of the laser was delivered over 83 s to cover the area of choroidal hyperpermeability, guided by ICGA images.

### ICGA and LSFG measurements

**Figure 5:**
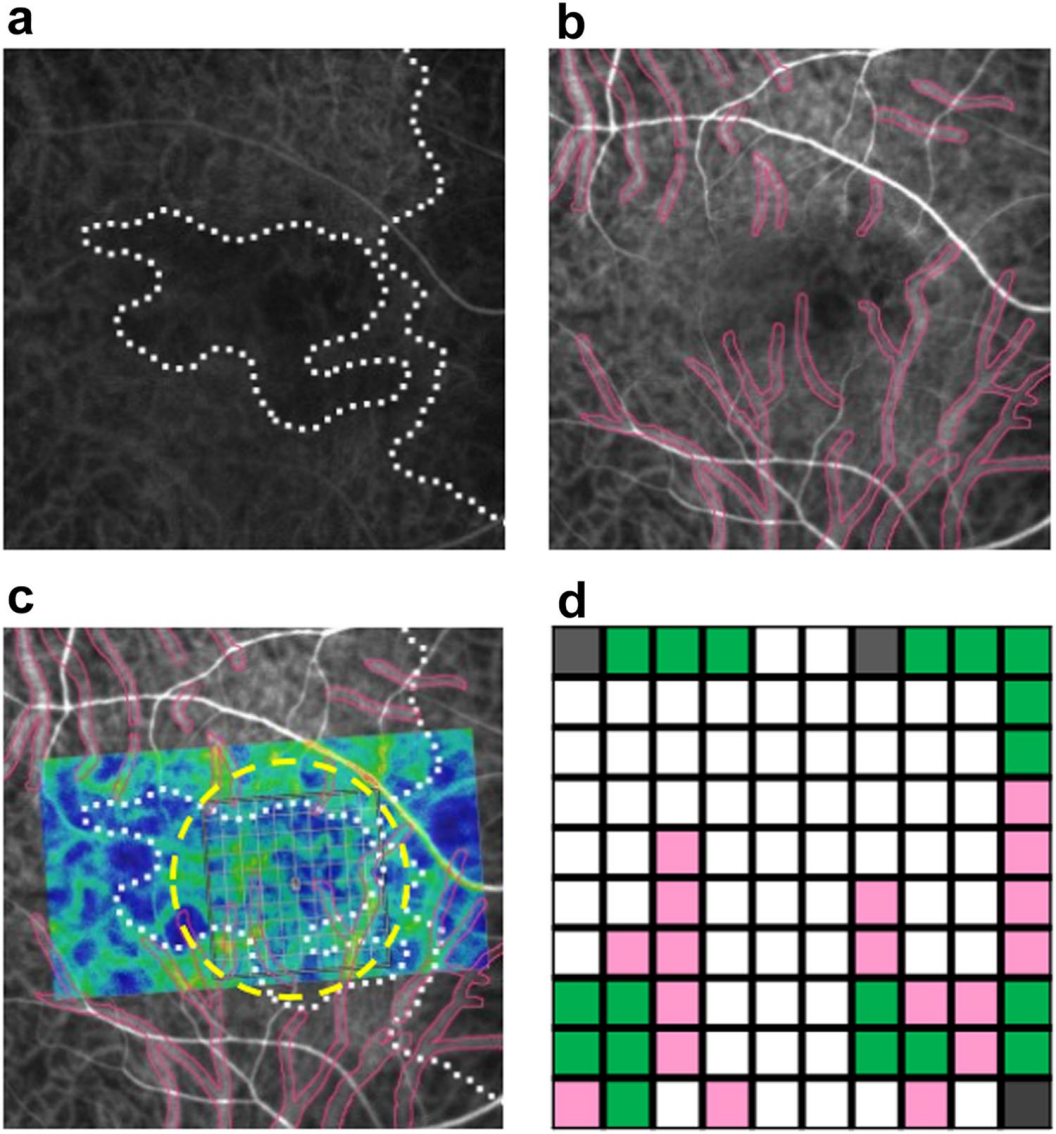
Analyses of laser-speckle flowgraphy (LSFG) segmented by pachyvessel area and filling delay area. (**a**) Choroidal filling delay was identified on early-phase indocyanine green angiography (ICGA) (white dotted lines). (**b**) Pachyvessels were identified on mid-phase ICGA if the vessel diameter was more than 150 μm. (**c**) A rubber band divided into 100 pieces was set inside both the area measured by LSFG (color composite map) and the photodynamic therapy (PDT) irradiation area (yellow circle). (**d**) Each grid square of the rubber band was labeled as the pachyvessel area (pink), filling delay area (white), or non-pachyvessel area (green). If a grid square was outside of the PDT irradiation area, the grid square was excluded from further analyses (gray).

The area of choroidal filling delay was detected in early phase ICGA (Fig. 5a). Pachyvessels with a vessel diameter greater than 150 µm on mid-phase ICGA were determined as the large choroidal veins in this study based on the recent report by Siihara et al. and the penetration ability of ICGA with wavelength of 790/830 nm (Fig. 5b)^30^. The LSFG system detected speckle patterns of 830-nm diode laser irradiation on the retina/choroid and analyzed a quantitative index of relative blood flow velocity and the MBR in arbitrary units. The built-in analysis software mathematically calculated the MBR and produced a composite color map of it (Fig. 5c). Using the LSFG analysis software (RubberBand EX Plugin ver. 1.0.10.0; Softcare), the rubber band (300 × 300 pixels) divided into 100 pieces was set in the PDT irradiation area to avoid atrophic lesions of the RPE (Fig. 5c). The rubber band was carefully set in the same area in each image by referring to retinal vessels on ICGA images. Each grid square with a side of 30 pixels was approximately equal to a side of 240 μm. Then, each grid square was labeled into three categories according to the presence of filling delay and pachyvessels: PV area, if a pachyvessel occupied more than 50% of the square; the FD area, if a pachyvessel vessel occupied less than 50% of the square and filling delay was observed in more than 50% of the square; and NPV area if both pachyvessel and filling delay occupied less than 50% of the area (Fig. 5d). If a grid square was outside the PDT irradiation area, the grid square was excluded from further analysis. If a grid square overlapped with an area with RPE atrophy, that grid square was excluded from further analysis. The MBR was measured three consecutive times in all eyes at each time point: before and 1, 3, and 6 months after half-dose PDT treatment, and the three consecutive data points in the same grid area were averaged. Then, the MBRs of each categorized area were averaged for further analyses. As the parameters for blood flow resistance, the skew and BOT were calculated based on the pulse waveform on LSFG and analyzed according to the same procedures as those of the MBR^31,32^.

### OCT measurements

Horizontal cross-sectional OCT images were used to measure the CCT and choroidal area. CCT was measured as the distance from the Bruch’s membrane to the chorioscleral border at the fovea. The choroidal area was measured using Niblack’s binarization method using ImageJ software (ImageJ ver. 2.0.0-rc-68/1.52 g, National Institutes of Health, Bethesda, MD), as previously reported^33,34^. The examined area was 3000 μm wide in the subfoveal choroid. The choroidal area was manually selected, extending from Bruch’s membrane to the chorioscleral border. The images were adjusted using the Niblack Auto Local threshold, and then the luminal and stromal areas were determined using the threshold tool. CVI was calculated as the ratio of the luminal area to the total choroidal area.

### Statistical analyses

All statistical analyses were performed using IBM SPSS (ver. 27; IBM Corp, Armonk, NY). Data represent mean ± standard deviation. Repeated ANOVA followed by Tukey’s test were performed in cases where the same subjects were evaluated at baseline, 1 month, 3 months, and 6 months. For the comparisons of groups, one-way ANOVA followed by Tukey’s test were performed, or otherwise indicated. Differences were considered significant at *P* < 0.05.

## Supplementary Information

Supplementary Information 1.

Supplementary Information 2.

## Data Availability

The datasets generated during and/or analyzed during the current study are available from the corresponding author on reasonable request.

## References

[1] Spaide RF (2022). Venous overload choroidopathy: A hypothetical framework for central serous chorioretinopathy and allied disorders. Prog. Retin. Eye Res..

[2] Lai TYY (2006). Safety enhanced photodynamic therapy with half dose verteporfin for chronic central serous chorioretinopathy: A short term pilot study. Br. J. Ophthalmol..

[3] Chan WM (2008). Safety enhanced photodynamic therapy for chronic central serous chorioretinopathy: One-year results of a prospective study. Retina.

[4] Fujita K (2015). One-year outcomes with half-dose verteporfin photodynamic therapy for chronic central serous chorioretinopathy. Ophthalmology.

[5] Oiwa K (2017). Half-dose photodynamic therapy for chronic central serous chorioretinopathy evaluated by focal macular electroretinograms. Jpn. J. Ophthalmol..

[6] Haga F (2017). Long-term prognostic factors of chronic central serous chorioretinopathy after half-dose photodynamic therapy: A 3-year follow-up study. PLoS ONE.

[7] Uetani R, Ito Y, Oiwa K, Ishikawa K, Terasaki H (2012). Half-dose vs one-third-dose photodynamic therapy for chronic central serous chorioretinopathy. Eye.

[8] Izumi T (2017). Structural analyses of choroid after half-dose verteporfin photodynamic therapy for central serous chorioretinopathy. Br. J. Ophthalmol..

[9] Iovino C (2020). Choroidal anatomic alterations after photodynamic therapy for chronic central serous chorioretinopathy: A multicenter study. Am. J. Ophthalmol..

[10] Ersoz MG, Arf S, Hocaoglu M, Sayman Muslubas I, Karacorlu M (2019). Patient characteristics and risk factors for central serous chorioretinopathy: An analysis of 811 patients. Br. J. Ophthalmol..

[11] Saito M (2015). Pulse waveform changes in macular choroidal hemodynamics with regression of acute central serous chorioretinopathy. Invest. Ophthalmol. Vis. Sci..

[12] Imanaga N (2021). Scleral thickness in central serous chorioretinopathy. Ophthalmol. Retina..

[13] Imamura Y, Fujiwara T, Margolis R, Spaide RF (2009). Enhanced depth imaging optical coherence tomography of the choroid in central serous chorioretinopathy. Retina.

[14] Agrawal R (2016). Choroidal vascularity index in central serous chorioretinopathy. Retina.

[15] Chung YR, Kim JW, Kim SW, Lee K (2016). Choroidal thickness in patients with central serous chorioretinopathy: Assessment of Haller and Sattler Layers. Retina.

[16] Matsumoto H (2020). Vortex vein anastomosis at the watershed in pachychoroid spectrum diseases. Ophthalmol. Retina..

[17] Saito M (2013). Macular choroidal blood flow velocity decreases with regression of acute central serous chorioretinopathy. Br. J. Ophthalmol..

[18] Saito M, Noda K, Saito W, Ishida S (2018). Relationship between choroidal blood flow velocity and choroidal thickness in patients with regression of acute central serous chorioretinopathy. Graefes Arch. Clin. Exp. Ophthalmol..

[19] Saito W, Hashimoto Y, Hirooka K, Ishida S (2020). Changes in choroidal blood flow velocity in patients diagnosed with central serous chorioretinopathy during follow-up for pachychoroid pigment epitheliopathy. Am. J. Ophthalmol. Case Rep..

[20] Kumashiro S (2021). Decrease in choroidal blood flow after half and one-third dose verteporfin photodynamic therapy for chronic central serous chorioretinopathy. BMC Ophthalmol..

[21] Savastano MC, Rispoli M, Savastano A, Lumbroso B (2015). En face optical coherence tomography for visualization of the choroid. Ophthalmic Surg. Lasers Imag. Retina..

[22] Wajer SD (2000). Velocity measurements of normal and sickle red blood cells in the rat retinal and choroidal vasculatures. Microvasc. Res..

[23] Schlotzer-Schrehardt U (2002). Dose-related structural effects of photodynamic therapy on choroidal and retinal structures of human eyes. Graefes Arch. Clin. Exp. Ophthalmol..

[24] Du W (2021). Dose-related structural effects of photodynamic therapy on rabbit choroidal structure. Ophthalmic Res..

[25] Ersoz MG, Karacorlu M, Arf S, Hocaoglu M, Sayman Muslubas I (2018). Pachychoroid pigment epitheliopathy in fellow eyes of patients with unilateral central serous chorioretinopathy. Br. J. Ophthalmol..

[26] Kantarci FA (2016). A pilot study of choroidal thickness in long-term smokers. Retina.

[27] Wei X (2019). Choroidal structural changes in smokers measured using choroidal vascularity index. Invest. Ophthalmol. Vis. Sci..

[28] Okawa K (2021). Correlation between choroidal structure and smoking in eyes with central serous chorioretinopathy. PLoS ONE.

[29] Isono H (2003). Observation of choroidal circulation using index of erythrocytic velocity. Arch. Ophthalmol..

[30] Shiihara H (2020). Quantitative analyses of diameter and running pattern of choroidal vessels in central serous chorioretinopathy by en face images. Sci. Rep..

[31] Shiga Y (2013). Waveform analysis of ocular blood flow and the early detection of normal tension glaucoma. Invest. Ophthalmol. Vis. Sci..

[32] Shiba T, Takahashi M, Hashimoto R, Matsumoto T, Hori Y (2016). Pulse waveform analysis in the optic nerve head circulation reflects systemic vascular resistance obtained via a Swan-Ganz catheter. Graefes Arch. Clin. Exp. Ophthalmol..

[33] Sonoda S (2014). Choroidal structure in normal eyes and after photodynamic therapy determined by binarization of optical coherence tomographic images. Invest. Ophthalmol. Vis. Sci..

[34] Mikoshiba Y (2018). A randomized clinical trial evaluating choroidal blood flow and morphology after conventional and pattern scan laser panretinal photocoagulation. Sci. Rep..

