# Supplementary material for: Choroidal hemodynamics in central serous chorioretinopathy after half-dose photodynamic therapy and the effects of smoking
Source: Sci Rep. 2022 Oct 11;12:17032. doi: 10.1038/s41598-022-21584-8 (PMC9553890; doi:10.1038/s41598-022-21584-8)
Supplement: Supplementary file 1 — Supplementary Information 1. [file 41598_2022_21584_MOESM1_ESM.pdf]

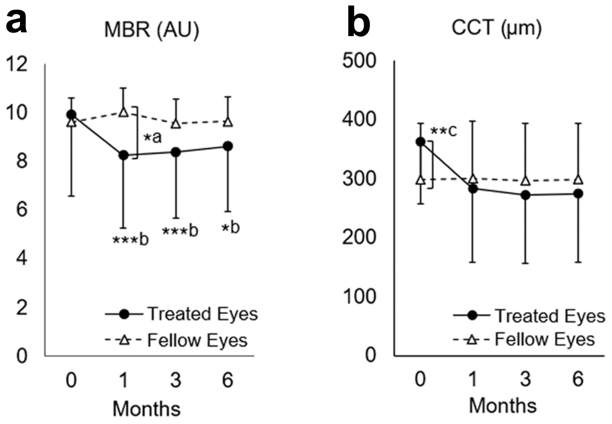

Supplementary Figure S1 Comparisons of mean blur rate (MBR) and central choroidal thickness (CCT) between eyes treated with half-dose photodynamic therapy (PDT) and their fellow eyes. (**a**) The MBR in the treated eyes significantly decreased after half-dose PDT. The MBR in the treated eyes was significantly lower than that in the fellow eyes 1 month after half-dose PDT. (**b**) The CCT in the treated eyes was significantly thicker than that in the fellow eyes at baseline. a: paired t-test; b: repeated analysis of variance followed by Tukey's test; c: Wilcoxon signed-rank test. AU: arbitrary units. *: $P < 0.05$, **: $P < 0.01$, ***: $P < 0.001$.
