# Supplementary material for: Choroidal hemodynamics in central serous chorioretinopathy after half-dose photodynamic therapy and the effects of smoking
Source: Sci Rep. 2022 Oct 11;12:17032. doi: 10.1038/s41598-022-21584-8 (PMC9553890; doi:10.1038/s41598-022-21584-8)
Supplement: Supplementary file 2 — Supplementary Information 2. [file 41598_2022_21584_MOESM2_ESM.pdf]

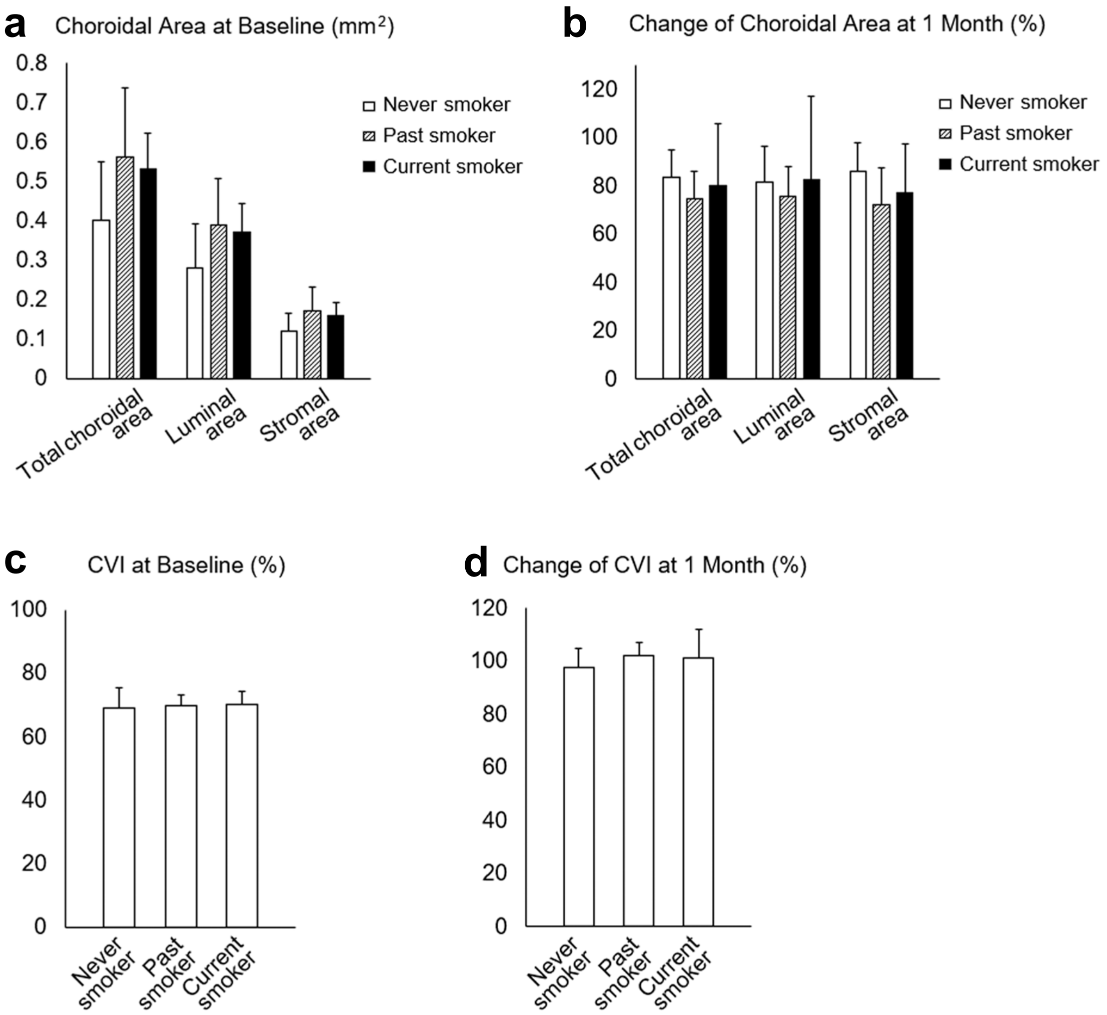

Supplementary Figure S2 Analyses of choroidal area according to smoking status. (**a**) Total choroidal, luminal, and stromal areas showed no significant difference among never, past, and current smokers at baseline. (**b**) The change ratio of each choroidal area 1 month after half-dose photodynamic therapy (PDT) was not significantly different according to smoking status. (**c, d**) Choroidal vascular index (CVI) at baseline (C) and change in CVI 1 month after half-dose PDT (D) showed no significant difference among never, past, and current smokers. One-way analysis of variance was performed for A–D, except for the stromal area of A for which Kruskal-Wallis test was performed.
